# COVID-19’s effect on healthcare disparities: delivery, reimbursement, and premature mortality in residentially segregated populations

**DOI:** 10.3389/fpubh.2025.1481814

**Published:** 2025-05-13

**Authors:** Kasabji Feras, Ferenc Vincze, Kinga Lakatos, Anita Pálinkás, László Kőrösi, László Ulicska, Karolina Kósa, János Sándor

**Affiliations:** ^1^Department of Public Health and Epidemiology, Faculty of Medicine, University of Debrecen, Debrecen, Hungary; ^2^Doctoral School of Health Sciences, University of Debrecen, Debrecen, Hungary; ^3^National Health Insurance Fund, Budapest, Hungary; ^4^Deputy State Secretariat for Social Inclusion, Ministry of Interior, Budapest, Hungary; ^5^Department of Behavioral Sciences, Faculty of Medicine, University of Debrecen, Debrecen, Hungary; ^6^ELKH-DE Public Health Research Group, Department of Public Health and Epidemiology, Faculty of Medicine, University of Debrecen, Debrecen, Hungary

**Keywords:** cross-sectional, COVID-19, segregation, inequality, healthcare, health reimbursement, general medical practitioner, Hungary

## Abstract

**Introduction:**

Spatially segregated, socio-economically deprived communities often face significant health disparities. This paper evaluates the impact of COVID-19 on healthcare delivery and reimbursement disparities in Hungary, particularly focusing on segregated populations.

**Aims:**

To examine healthcare utilization and reimbursement patterns among patients in segregated areas (SA) and non-segregated or complementary areas (CA) during the first year of the COVID-19 pandemic, compared to pre-pandemic levels, and to understand how these patterns influenced overall health outcomes.

**Methods:**

A cross-sectional study using 2019 and 2020 healthcare data from all Hungarian general medical practices (GMPs) was conducted. Segregated areas were identified based on governmental criteria, and healthcare indicators were standardized by age, sex, and socioeconomic status. Key indicators included General Practitioner (GP) visits, outpatient services, Magnetic Resonance Imaging (MRI) and Computed Tomography (CT) usage, hospitalizations, healthcare reimbursement, and premature mortality.

**Results:**

In 2020, there was a notable reduction in healthcare services utilization due to COVID-19 restrictions, with GP visits declining by 10.43% in SAs and 4.13% in CAs. Outpatient services decreased by 19.16% in SAs and 12.45% in CAs, while hospitalizations dropped by over 23.52%. Despite these reductions, the relative risk (RR) of healthcare service use remained higher in SAs compared to CAs (RR = 1.22, 95% CI: 1.219;1.223). Healthcare reimbursement was significantly lower in SAs (RR = 0.940, 95% CI: 0.929;0.951), and premature mortality was higher (RR = 1.184, 95% CI: 1.087;1.289).

**Conclusion:**

The COVID-19 pandemic led to a significant reduction in healthcare utilization across Hungary. However, segregated populations in 2020 continued to have higher healthcare service use but received lower reimbursement, indicating persistent healthcare disparities. The consistently higher premature mortality rate in SAs underscores the need for targeted interventions and improved healthcare access and quality for vulnerable communities. Future policies should be built on data from comprehensive monitoring systems to address and mitigate these disparities, ensuring equitable healthcare access in and out of health crises.

## 1 Introduction

Recently, there has been a growing scientific focus on the overarching subject of residential segregation and its impact on human health. This includes a specific examination of the effects of ethnic segregation on health outcomes in minorities, particularly in the context of the COVID-19 pandemic (1, 2). Several studies have unequivocally demonstrated that there are health disparities between individuals residing in segregated areas and those in nonsegregated areas; these disparities encompass but are not restricted to premature mortality and exposure to both communicable and noncommunicable disease risk factors (3, 4).

The COVID-19 pandemic has exacerbated existing health disparities, disproportionately affecting racial and ethnic minority groups in the United States (5). Data from the Centers for Disease Control and Prevention (CDC) (6) reveal that segregated ethnic populations have experienced higher rates of infection, hospitalization, and death than their counterparts. These disparities were corroborated by further findings in European studies (7, 8), in which it was often found that those living in segregated, low socio economic status (SES) neighborhoods had disproportionately higher rates of COVID-19.

This disparity was attributed to several factors, including preexisting health conditions, socioeconomic challenges, and limited access to quality healthcare (9). More specifically, a study revealed that, in minority communities, living in overcrowded areas and having low levels of education were linked to higher infection rates, whereas higher employment rates and living in communities with public facilities such as open spaces and recreational areas had the opposite effect, which further exacerbates their already adversely affected health status (10).

Moreover, the COVID-19 pandemic has highlighted significant variations in how European countries monitor and manage the impact of major health threats on primary healthcare (PHC) services. According to the Eurodata Study (11), these differences in disease surveillance and healthcare service utilization underscore the need for a unified and comprehensive approach to monitoring pandemic indicators and their effects on PHC systems.

In Hungary, in January 2020, the government preemptively formed an 11-member operative corps led by the interior minister and the minister of human capacities, and they were tasked with organizing medical and epidemiological measures in response to the COVID-19 pandemic, including testing and the allocation of personnel and physical resources (12).

After the first case of COVID-19 was discovered on March 4, 2020, several measures were implemented to combat the spread of the disease, including a general lockdown between March and June, travel restrictions, social distancing mandates, and the closure of nonessential businesses and education facilities. Further restrictions were applied during the second and third waves in November 2020, which softened only around December 2021 (13). Owing to the increased number of infections during the pandemic, primary and specialized healthcare services were restricted to providing more pandemic-focused patient care and vaccinations. The full effects of these measures on the overall health status of the population have not been well documented, especially with respect to vulnerable populations such as segregated minorities.

Our previous study in Hungary demonstrated that healthcare services were more commonly used during the onset of the COVID-19 pandemic in 2020 by individuals living in segregated areas (SAs) than by those living in nonsegregated or complementary areas (CAs). This trend was associated with a lower quality of care, as indicated by the reduced amount of healthcare reimbursement (14). Specifically, the decrease in reimbursement among SA patients for outpatient services and advanced imaging tools such as computed tomography or magnetic resonance imaging (MRI/CT), which are essential tools for the diagnosis, management, and treatment of diseases, likely contributed to illnesses remaining undetected until their condition worsened and required hospitalization.

The trend of higher GP visits and hospitalization rates among Hungarian Roma was evident prior to Covid-19 according to a study conducted in 2018 (15), further evidence shows a similar trend among minorities, migrants and other vulnerable groups in the EU and US long before Covid/19, demonstrating higher inpatient care utilization and lower engagement with preventive and mental healthcare services as well as access to certain medications and vaccines (16–20).

This analysis aims to explore the change of segregation related disparities in healthcare use, comparing the pandemic health care use to the prepandemic patterns within SA and CA populations. Addressing these disparities requires a multifaceted approach that includes improving access to healthcare, tackling social determinants of health, and ensuring equitable distribution of resources such as vaccines and testing.

## 2 Methods

### 2.1 Setting

The study utilized individual-level health records collected from January 1st to December 31st, 2019, prior to the COVID-19 pandemic, and from the same period during the first year of the pandemic in 2020. Aggregated indicators were assessed at the level of general medical practices (GMPs). All GMPs (*N* = 4,359) providing care to adults in Hungary were included. Each GMP was contracted with the National Health Insurance Fund of Hungary (NHIF), the sole agency financing health insurance in Hungary. Which provided data for secondary analysis of healthcare utilization, reimbursement, and the health status of adults.

### 2.2 Design

A cross-sectional study was conducted with Hungarian GMPs serving segregated adults. Segregated areas (SAs) were identified via the classification outlined in the governmental decree 314/2012 (XI.8.) (21), which defined SAs as settlement clusters where a significant proportion of adults aged 15–59 had no earned income and only a primary-level education, reflecting socioeconomic deprivation according to Census data. The NHIF classified each Hungarian household as being in either an SA or a nonsegregated area (complementary area: CA), and these categories were mutually exclusive. Using addresses, any adult aged 18 years and above could thus be categorized as residing in an SA or a CA. GMPs without patients residing in an SA were excluded from the analysis.

#### 2.2.1 Outcome indicators

##### 2.2.1.1 Healthcare delivery

The rates of healthcare delivery for various services over the preceding 12 months were calculated as the number of patients utilizing the service per number of patients affiliated with a GMP at the end of each year. These indicators included: (1) the number of GP visits, (2) outpatient service use, (3) the number of uses of MRI/CT services, and (4) the number of hospitalizations (Table 1).

**Table 1:** Indicators for health care utilization and for premature mortality.

Indicator name	Indicator definition	Calculation method
Number of GP visits	Number of GP-patient encounters	Total number of GP visits in the previous 12 months/the number of adults affiliated to the GMP
Outpatient service use	Use of outpatient specialist care, excluding CT and MRI examinations	Number of episodes**/**insured persons registered with a general practitioner during the previous 12 months
Number of MRI/CT use	CT and MRI examinations used as part of outpatient specialist care	Number of episodes**/**insured persons registered with a general practitioner during the previous 12 months
Number of hospitalizations	Use of inpatient specialist care	Number of episodes**/**insured persons registered with a general practitioner during the previous 12 months
Total healthcare utilization	Total number of healthcare service uses from all indicators	Addition of all episodes of healthcare utilization across all indicators
Outpatient service reimbursement	Reimbursement for outpatient specialist care, with the exception of CT and MRI examinations	Health insurance payments**/**insured persons registered with a general practitioner during the previous 12 months
MRI/CT reimbursement	Reimbursement for CT and MRI examinations used as part of outpatient specialist care	Health insurance payments**/**insured persons registered with a general practitioner during the previous 12 months
Hospitalization reimbursement	Reimbursement of inpatient specialist care	Health insurance payments**/**insured persons registered with a general practitioner during the previous 12 months
Medication reimbursement	Health insurance drug expenditures	Total amount reimbursed**/**insured persons registered with a general practitioner during the previous 12 months
Total healthcare reimbursement	Total amount of money paid per capita for all healthcare services uses	Total amount reimbursed/insured persons registered with a general practitioner during the previous 12 months for all indicators
Premature mortality	All deaths of adults aged <65 and have not changed their GP in 5 years	Total number of deaths in the previous 12 months/the number of adults affiliated to the GMP

##### 2.2.1.2 Healthcare reimbursement

The average health insurance expenditures (converted to Euro using a conversion rate of 383.18 HUF/EUR) per capita for the investigated services and for medication reimbursement were computed for each GMP over the preceding 12 months. Basic GMP financing was not included among the reimbursement indicators, as it does not affect the variability in the average per capita financing, given that the NHIF finances GMPs per capita regardless of the number of patient visits (Table 1).

##### 2.2.1.3 Premature mortality

All-cause premature mortality was defined as all deaths of adults under the age of 65 who had not changed their GMP in the past 5 years. This criterion was applied to exclude individuals who died under the care of a new GMP with whom they had not previously interacted regarding their health (Table 1).

### 2.3 Statistical analysis

The outcome indicators of a GMP were computed for the associated SA and CA. Indicators were indirectly standardized by age (age groups: 18–24, 25–29, 30–34, 35–39, 40–44, 45–49, 50–54, 55–59, 60–64, 65–69, 70–74, 75–79, and 80 and above) and sex, as well as eligibility for an exemption certificate. (Exemption certificates, issued by local municipalities, are granted to patients with disadvantaged socioeconomic status and chronic diseases if recommended by GPs and ensure free access to medicines and medical devices.) If a GMP provided care to multiple areas, the observed and expected values were aggregated to obtain GMP-specific SA and CA measures. The total healthcare utilization and insurance reimbursement indicators were calculated from the total observed and expected values for each GMP.

The division of the GMP-level observed values by the expected values, using the stratum-specific national average as a reference, yielded standardized risk ratios for SAs (SRsa) and CAs (SRca) for each indicator and GMP. These GMP-level data were further aggregated to derive country-level standardized measures for SAs and CAs.

The relative risk (RR) in SAs was represented by the risk ratio, which was calculated as the ratio of the SRsa to the SRca for each GMP, along with corresponding 95% confidence intervals (95% CIs). The GMP-level data were aggregated to compute the SRsa and SRca and RR for the entire country.

Impact measures, such as excess cases in SAs, the percentage of risk attributable to segregation in the population in SAs (attributable risk, AR), and the percentage of risk attributable to segregation in the population in the entire country (population attributable risk) were also computed via standardized ratios.

The standardized measures observed in 2020 were compared with those from 2019 by 95% CIs.

The data analysis was performed with SPSS version 20 (IBM Corporation, New York, NY, United States).

### 2.4 Ethics permission

The Hungarian NHIF provided the data used in this study. In our secondary analysis, ethics approval and written informed consent were not required since all utilized data were aggregated geographically in accordance with the Hungarian legal framework. The methodology used to create segregation-specific indicators was approved by the Office of the Commissioner for Fundamental Rights (AJB-3147/2013), the general director of the National Health Insurance Fund (NHIF) (E0101/215–3/2014), and the Hungarian National Authority for Data Protection and Freedom of Information (NAIH/2015/826/7 N).

## 3 Results

### 3.1 Demographics

**Figure 1:**
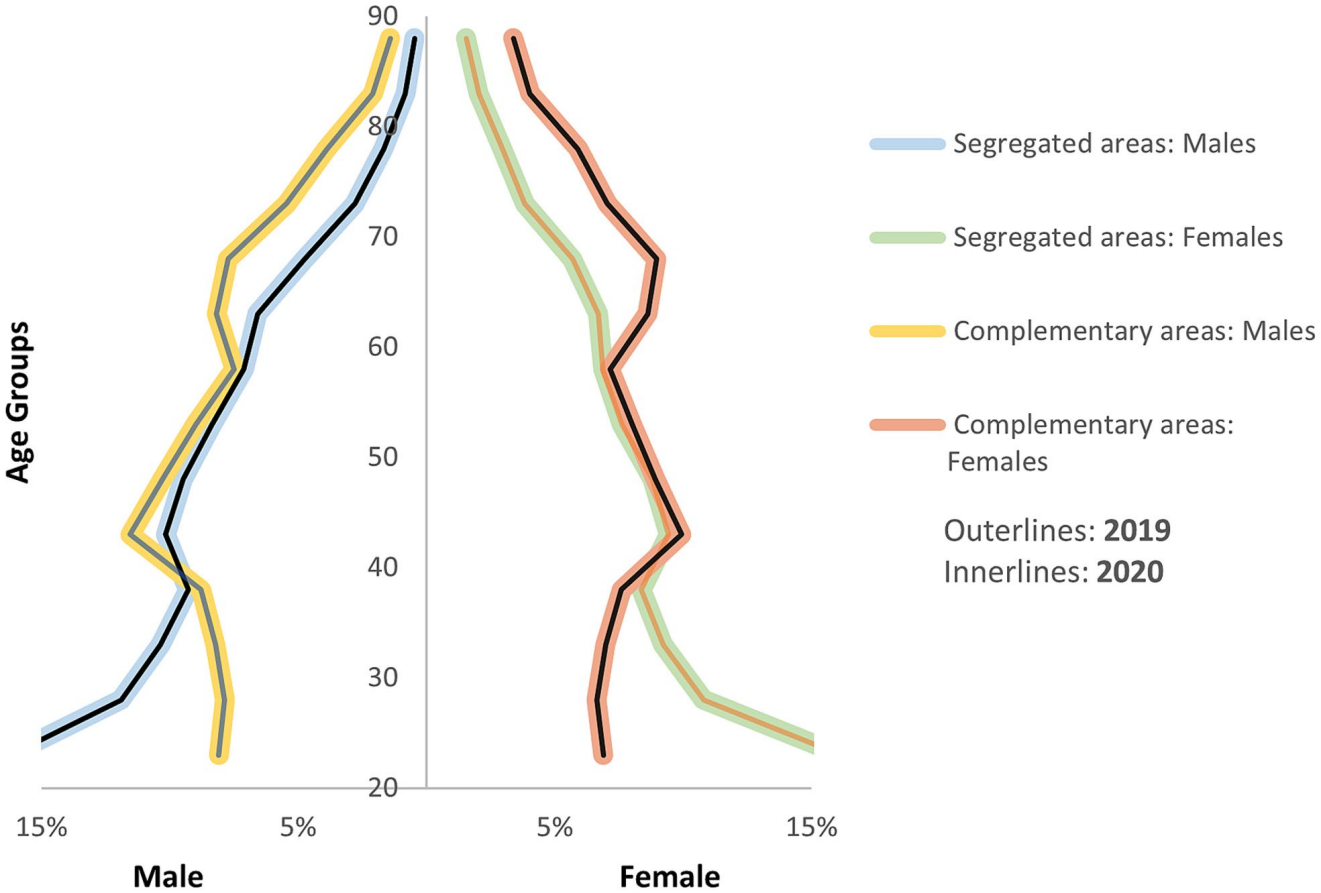
Population pyramid of complementary and segregated areas in 2019 and 2020.

According to our data, there was no significant change in the studied population between 2019 and 2020. In 2020, our study population consisted of 7,385,641 adults (3,456,560 men and 3,929,081 women), an increase of 1729 adults compared with 2019 primarily due to demographic transition. The 2,071 segregated areas identified in 2020 by the government included according to our research 283,876 adults (139,507 men; 144,369 women), an increase of only 4 males and 3 females from the 2019 population. Similarly, the demographic structure of both communities remained similar between the 2 years, with notable differences between the SA and CA populations (Figure 1).

The mean age of the SA population (total: 43.3 years; men: 42.2 years; women: 44.4 years) was lower than that of the CA population (total: 50.4 years; men: 48.5 years; women: 52.1 years) This difference is largely attributable to higher fertility rates in SA populations, approximately 2 to 3.5 times higher than in CA populations (22), as well as worse health outcomes that contribute to a life expectancy, approximately 10 years lower than in CAs (23, 24). These factors result in a relatively younger population in SAs. Additionally, the older adult dependency ratio (the percentage of the older adult population aged above 65 years compared with those aged 15 years and above) remained markedly lower in SAs (15.4%) than in CAs (33.7%).

### 3.2 Health care use in the pandemic period

According to the crude measures, in Hungary, there was a notable decrease in healthcare service utilization in 2020 compared with that in 2019 (Table 2). The number of GP visits declined by 10.43% in SAs and 4.13% in CAs, whereas outpatient service use decreased by 19.16% in SAs and 12.45% in CAs. The highest observed reduction was in the number of hospitalizations, which decreased by more than 23.52% followed by that in MRI/CT use, which decreased by 17.82%, especially among segregated patients. Reimbursement for these services also declined, except for medication reimbursement, which increased by 7.24% in SAs and 3.78% in CAs. Premature mortality increased in CAs and decreased in SAs (details in Supplementary Table S1).

**Table 2:** Restriction to the healthcare system in 2020 due to COVID-19 pandemic in the segregated (SA) and complementary areas (CA) of the study population compared with 2019.

Indicator	Change in SAs	Change in CAs	Total change
Number of GP visits	−10.43%	−4.13%	−4.41%
Outpatient service use	−19.16%	−12.45%	−12.69%
Number of MRI/CT use	−21.73%	−17.69%	−17.82%
Number of Hospitalizations	−24.22%	−23.49%	−23.52%
Outpatient service reimbursement	−14.35%	−15.99%	−15.90%
MRI/CT reimbursement	−5.76%	−7.65%	−7.53%
Hospitalization reimbursement	−13.64%	−18.27%	−18.09%
Medication reimbursement	7.24%	3.78%	3.92%
Premature mortality	−1.39%	2.69%	2.46%

### 3.3 Healthcare delivery

**Figure 2:**
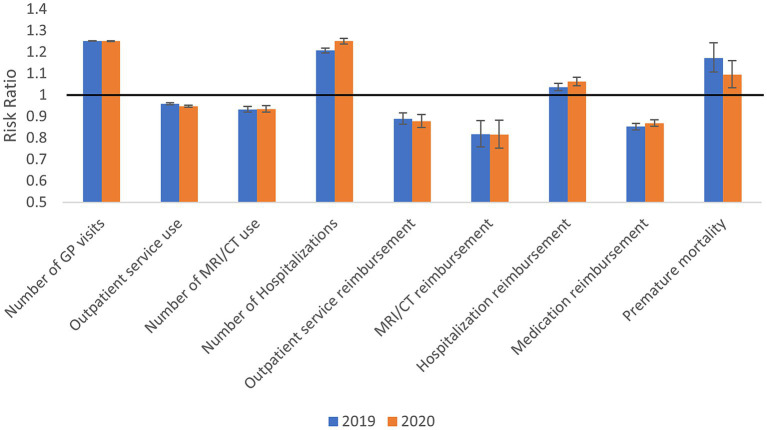
Standardized relative health care use and premature mortality (relative risk with 95% confidence interval) in the segregated areas compared with complementary areas in 2019 and 2020.

With respect to GP visits, as illustrated by the changes in RR in Figure 2, the inequality remained unchanged from 2019 to 2020 (RR = 1.251 95% CI: 1.249; 1.253). Moreover, in both years, GP visits were more frequent among segregated patients than among nonsegregated patients. The number of excess cases was highest in 2019, before COVID-19, and decreased significantly in 2020, decreasing from 446,734.538 (95% CI: 444,395.971; 449,070.034) to 400,024.581 (95% CI: 397,811.133; 402,234.958). However, the attributable risk remained the same before and after COVID-19, with an AR of 20.1% (95% CI: 20.0%; 20.2%) (Tables 3, 4).

**Table 3:** Comparison of healthcare utilization and reimbursement between segregated and complementary areas among Hungarian adults in 2019 and 2020.

Indicators	Year	Total	Segregated areas		Complementary areas		Relative risk in segregated areas [95%CI]
		N	N	Standardized risk ratios [95%CI*]	N	Standardized risk ratios [95%CI]	
Healthcare delivery (episodes)							
GP visits	2019	49,959,120	2,225,524	1.238 [1.236; 1.239]	47,733,596	0.989 [0.989; 0.990]	1.251 [1.249; 1.253]
	2020	47,754,032	1,993,344	1.238 [1.237; 1.240]	45,760,688	0.990 [0.989; 0.990]	1.251 [1.249; 1.253]
Use of outpatient services	2019	5,180,428	186,065	0.961 [0.956; 0.965]	4,994,363	1.002 [1.001; 1.003]	0.959 [0.954; 0.963]
	2020	4,522,976	150,414	0.951 [0.946; 0.956]	4,372,562	1.003 [1.002; 1.004]	0.948 [0.943; 0.953]
Use of MRI/CT	2019	600,585	19,264	0.937 [0.923; 0.950]	581,321	1.004 [1.002; 1.007]	0.933 [0.919; 0.946]
	2020	493,566	15,078	0.940 [0.925; 0.955]	478,488	1.005 [1.002; 1.008]	0.935 [0.920; 0.950]
Use of hospital service	2019	1,094,222	46,882	1.199 [1.188; 1.210]	1,047,340	0.993 [0.991; 0.995]	1.207 [1.196; 1.218]
	2020	836,818	35,527	1.241 [1.228; 1.254]	801,291	0.992 [0.990; 0.994]	1.250 [1.237; 1.264]
Total healthcare utilization	2019	56,834,355	2,477,735	1.208 [1.206; 1.209]	54,356,620	0.991 [0.990; 0.991]	1.219 [1.218; 1.221]
	2020	53,607,392	2,194,363	1.211 [1.209; 1.212]	51,413,029	0.991 [0.991; 0.991]	1.222 [1.220; 1.223]
Healthcare reimbursement (Euro per capita)							
Outpatient service	2019	50.73	41.22	0.896 [0.869; 0.922]	51.12	1.006 [1.001; 1.012]	0.890 [0.863; 0.917]
	2020	42.67	35.31	0.885 [0.856; 0.915]	42.94	1.008 [1.002; 1.014]	0.878 [0.848; 0.908]
MRI/CT	2019	9.27	6.73	0.823 [0.765; 0.886]	9.37	1.008 [0.996; 1.021]	0.817 [0.758; 0.880]
	2020	8.57	6.34	0.823 [0.760; 0.890]	8.65	1.009 [0.996; 1.022]	0.815 [0.752; 0.883]
Hospital service	2019	152.17	135.89	1.036 [1.019; 1.053]	152.82	0.999 [0.996; 1.002]	1.037 [1.020; 1.054]
	2020	124.63	117.36	1.062 [1.043; 1.082]	124.91	0.999 [0.996; 1.003]	1.063 [1.043; 1.083]
Medications	2019	141.62	121.23	0.857 [0.842; 0.872]	142.44	1.006 [1.002; 1.009]	0.852 [0.837; 0.867]
	2020	147.18	130.01	0.871 [0.856; 0.887]	147.82	1.003 [0.999; 1.006]	0.869 [0.854; 0.884]
Total healthcare reimbursement	2019	353.79	305.07	0.933 [0.923; 0.944]	355.75	1.003 [1.001; 1.005]	0.931 [0.920; 0.941]
	2020	323.05	289.02	0.940 [0.929; 0.952]	628.96	1.002 [1.000; 1.004]	0.938 [0.927; 0.950]
Premature mortality							
Premature mortality	2019	21,005	1,225	1.162 [1.098; 1.229]	19,780	0.991 [0.977; 1.005]	1.172 [1.107; 1.242]
	2020	21,521	1,208	1.088 [1.028; 1.151]	20,313	0.994 [0.980; 1.008]	1.095 [1.033; 1.160]

*95%CI: 95% confidence interval.

**Table 4:** The impact of segregation on healthcare performance among Hungarian adults in 2019 and 2020.

Indicators	Year	Excess [95%CI] ^#^	Attributable risk [95%CI]^#^	Population attributable risk
Healthcare delivery (episodes)				
GP visits	2019	446,735 [444,396; 449,070]	20.1% [20.0%; 20.2%]	0.894%
	2020	400,025 [397,812; 402,235]	20.1% [20.0%; 20.2%]	0.838%
Use of outpatient services	2019	−8,023 [−8,907; −7,143]	−4.3% [−4.8%; −3.8%]	−0.155%
	2020	−8,242 [−9,045; −7,442]	−5.5% [−6.0%; −4.9%]	−0.182%
Use of MRI/CT	2019	−1,394 [−1,688; −1,104]	−7.2% [−8.8%; −5.7%]	−0.232%
	2020	−1,047 [−1,306; −0.791]	−6.9% [−8.7%; −5.2%]	−0.212%
Use of hospital service	2019	8,047 [7,694; 8,397]	17.2% [16.4%; 17.9%]	0.735%
	2020	7,117 [6,820; 7,410]	20.0% [19.2%; 20.9%]	0.850%
Total healthcare utilization	2019	445,440 [442,908; 447,969]	18.0% [17.9%; 18.1%]	0.784%
	2020	397,922 [395,543; 400,297]	18.1% [18.0%; 18.2%]	0.742%
Healthcare reimbursement (Euro per capita)				
Outpatient service	2019	−5.6 [−5.6; −5.6]	−12.4% [−15.8%; −9.1%]	−0.389%
	2020	−4.9 [−5.0; −4.9]	−14.0% [−17.8%; −10.2%]	−0.418%
MRI/CT	2019	−1.7 [−1.7; −1.6]	−22.5% [−31.8%; −13.8%]	−0.630%
	2020	−1.4 [−1.4; −1.4]	−22.7% [−32.8%; −13.3%]	−0.607%
Hospital service	2019	5.3 [5.2; 5.3]	3.5% [1.9%; 5.1%]	0.122%
	2020	7.0 [6.9; 7.0]	5.9% [4.2%; 7.7%]	0.203%
Medications	2019	−23.1 [−23.2; −23.1]	−17.3% [−19.4%; −15.3%]	−0.574%
	2020	−19.6 [−19.7; −19.6]	−15.1% [−17.1%; −13.1%]	−0.483%
Total healthcare reimbursement	2019	−25.0 [−25.1; −25.0]	−7.5% [−8.6%; −6.3%]	−0.249%
	2020	−19.0 [−19.1; −18.9]	−6.6% [−7.8%; −5.3%]	−0.213%
Premature mortality				
Premature mortality	2019	180.0 [119.8; 236.9]	14.7% [9.8%; 19.3%]	0.857%
	2020	104.4 [40.4; 164.9]	8.6% [3.3%; 13.7%]	0.485%

^#^95%CI, 95% confidence interval.

In contrast to the results for GP visits, the inequality in outpatient use was lowest in 2019, before COVID-19 (RR = 0.959, 95% CI: 0.954; 0.963), and increased to 0.948 (95% CI: 0.943; 0.953) in 2020. The SA populations used services less often than the CA populations did in both years. The attributable risk significantly increased between 2019 and 2020: −4.3% (95% CI: −4.8%; −3.8%) and −5.5% (95% CI: −6.0%; −4.9%), respectively. The number of excess cases did not significantly differ between 2019 and 2020 (Table 4).

With respect to the use of CT/MRI services, SA patients utilized these services less often than CA patients did in both years. There was no significant difference in inequality between 2019 and 2020 (Figure 2). Furthermore, no significant difference in excess cases or attributable risk between 2019 and 2020 was found. Our study revealed a notable shift in hospital service utilization and total healthcare utilization between 2019 and 2020, particularly in the context of the COVID-19 pandemic. The inequality increased significantly from 2019 to 2020 (RR = 1.207, 95% CI: 1.196–1.218) (RR = 1.250, 95% CI: 1.237–1.264). In both years, segregated patients were hospitalized more often than nonsegregated patients (Table 3). The attributable risk was greater during the first year of the COVID-19 pandemic (AR = 20.0, 95% CI: 19.2%; 20.9%) than in 2019 (AR = 17.2, 95% CI: 16.4%; 17.9%). However, the number of excess cases was higher in 2019 than in 2020 (excess: 8046.964, 95% CI: 7693.827; 8396.919) (Table 4).

In general, regarding total healthcare utilization patterns, there was no significant difference between SA and CA patients (RR = 1.219, 95% CI: 1.218; 1.221) in 2019 and (RR = 1.222, 95% CI: 1.220; 1.223) in 2020 (Table 3). The attributable risk for segregated patients’ healthcare services remained similar in 2019 (18.0, 95% CI: 17.9%; 18.1%) and 2020 (18.1, 95% CI, 18.0%; 18.2%), whereas excess cases were higher in 2019 (445,440.419, 95% CI: 442,908.290; 447,969.396) than in 2020 (397,921.780, 95% CI: 395,543.285; 400,297.130) (Table 4).

### 3.4 Healthcare reimbursement

Overall, the trend shows a clear inequality between segregated and nonsegregated patients regarding reimbursement to GMPs who received reduced payments when treating segregated patients according to the total healthcare reimbursement risk ratio. However, the COVID-19 pandemic had no significant effect on this trend (Figure 2). GMPs were reimbursed less than expected for providing outpatient services to SA patients than for providing those services to CA patients in both years. The inequality was not significantly different between 2019 and 2020 (RR = 0.890, 95% CI: 0.863; 0.917 and RR = 0.878, 95% CI: 0.848; 0.908, respectively) (Table 3). In all years, GMPs received reduced payments for treating segregated patients, but there was no significant difference between the years in terms of excess cases. No difference in attributable risk was found due to the COVID-19 pandemic (Table 4).

With respect to MRI/CT services, SA patients were associated with lower GP reimbursement than CA patients were. Moreover, this service exhibited the most significant disparity in terms of GMP payments in both 2019 (RR = 0.817, 95% CI: 0.758; 0.880) and 2020 (RR = 0.815, 95% CI: 0.752; 0.883), with no significant differences found between the years for RR, AR, and excess cases. However, GMPs received significantly higher reimbursement for hospital services for SA patients than for CA patients (Table 3).

### 3.5 Premature mortality

Compared with nonsegregated individuals, segregated patients had greater premature mortality rates in both 2019 and 2020, with RRs of 1.172 (95% CI: 1.107; 1.242) and 1.095 (95% CI: 1.033; 1.160), respectively (Figure 2). However, there was no significant change in the disparity in premature mortality between the SA and CA groups during the COVID-19 pandemic (Table 3).

## 4 Discussion

This study examined the impact of the COVID-19 pandemic-induced healthcare restrictions implemented by the Hungarian government at the beginning of 2020 on disparities in residential segregation-related healthcare utilization, which existed before the pandemic.

Restrictions implemented during the pandemic aimed at minimizing virus transmission, limited non-essential consultations. This approach prioritized COVID-19 cases and reduced exposure for both patients and healthcare providers which led to sizeable reductions in healthcare use and reimbursement across each investigated sector, ranging from a 4.41% reduction in the number of GP visits to a 23.52% reduction in hospitalizations. While medication reimbursement increased by 3.92% likely due to an expanded reliance on telemedicine and remote prescriptions to maintain continuity of care. This increase in prescription reimbursement aligns with Hungary’s accelerated adoption of telemedicine during the pandemic, as noted in national healthcare surveys (25). Overall, these measures were accompanied by a 2.46%, increase in premature mortality. However, these trends were not unique to Hungary; healthcare access was found to be similarly restricted across the EU with regard to hospitalization (26), the use of outpatient services and advanced diagnostics (27), and psychiatric treatments (28). While research on the effects of the COVID-19 pandemic on healthcare access among vulnerable groups was initially limited, recent studies have increasingly addressed this issue. For example, research conducted in the Netherlands has revealed that vulnerable populations experienced up to a 10% decrease in access to health services and general practitioners (29, 30). Furthermore, a systematic review encompassing 19 studies across the EU highlighted the inequalities experienced by deprived groups with regard to access to healthcare services due to their disadvantaged socioeconomic status (31). Despite this overall reduction in healthcare utilization, our investigation revealed that segregation-related inequalities did not differ significantly between 2019 and 2020 suggesting persistent barriers to healthcare among SA patients. There was a preexisting underuse of outpatient care services (RR_2019_ = 0.959, 95% CI: 0.954; 0.963; RR_2020_ = 0.948, 95% CI: 0.943; 0.953) and an overuse of hospitals (RR_2019_ = 1.207, 95% CI: 1.196; 1.218; RR_2020_ = 1.250, 95% CI: 1.237; 1.264) in SAs compared with in CAs. Although these minor changes (1.1% in outpatient services and 4.3% in hospital use) were statistically significant due to the large size of the investigated population, they had negligible public health importance. With respect to healthcare reimbursement, the RRs for each indicator were consistent in both 2019 and 2020.

The RR for premature mortality was consistently greater among SA residents than among CA residents in both 2019 and 2020, with no significant change. Previous studies, regardless of whether they took into account the COVID-19 pandemic, have demonstrated the significant impact of segregation on mortality outcomes (3, 4). Living in segregated areas is associated with barriers to accessing quality healthcare, as indicated by global research (32, 33). These barriers may contribute to worse health outcomes and ultimately result in higher mortality rates. The lack of a significant difference in the disparity in premature mortality between the SA and CA populations before and during the COVID-19 pandemic suggests that these disparities persisted regardless of presence of a global health threat.

While the restrictions related to the COVID-19 pandemic implemented in 2020 in Hungary decreased overall healthcare service utilization, they did not disproportionately affect segregated populations in terms of accessing healthcare services, as evident by the nonsignificant difference in healthcare utilization patterns between the years, as well as the unchanged mortality gap.

Moreover, settlement-level aggregated COVID-19 mortality in Hungary was inversely associated with the settlements’ socioeconomic status. This status was computed based on factors such as income, education, unemployment, family size, housing density, and car ownership. The higher mortality rates were due mainly to the significant excess number of cases among older adult individuals above 65 years in deprived areas, whereas individuals younger than 65 years experienced a less pronounced effect (34). Despite this, a 2021 cross-sectional Hungarian survey, which is part of the International Social Survey Program, indicated no associations between GP visits or hospitalization and education level (35). However, lower education levels have been shown to be linked to less frequent implementation of epidemiologic measures such as testing for SARS-CoV-2 infection, contact tracing, and vaccination (36). Our findings confirm that COVID-19-related healthcare restrictions did not exacerbate the socioeconomic healthcare inequalities that already existed, but we could not verify the small excess mortality among younger adults in deprived populations. This discrepancy may be attributed to the varying impacts of socioeconomic indicators and residential segregation. These findings suggest that SES-based inequalities in the implementation of measures intended to control the pandemic, rather than healthcare use, were responsible for the socioeconomic gradient in COVID-19 mortality. This hypothesis requires further investigation.

The findings of this study in the Hungarian population are not in accordance with those regarding the mainstream international experience. Recent papers reported that by reducing healthcare access, the COVID-19 pandemic has worsened known disparities in already vulnerable communities, exacerbating their ill health and increasing mortality (2, 37, 38).

### 4.1 Strengths and limitations

A key advantage of our study is the consistent quality of the data used across both years, which was guaranteed by the standardized NHIF protocols for data collection (39). Furthermore, our investigation included all Hungarian adults living in SAs and CAs with detailed health use and reimbursement data, since all Hungarian GMPs are required to be registered with the NHIF, therefore eliminating the potential for selection bias and maximizing statistical power.

Our study did not adjust the healthcare use indicators for the health status and healthcare needs of the investigated populations. The relatively unhealthy living conditions, lifestyle and poor health status of segregated populations might partially explain the observed overuse of GP services and hospitalizations in SAs. However, it does not explain the underuse of outpatient and CT/MRI services. Moreover, these observed underutilizations should be evaluated as underestimations of the seriousness of the real inequality, since the reference level for needs is higher in SAs.

While this study focuses on healthcare services, it is important to note that other public services, such as social support and community outreach, also impact well-being in segregated areas. Future research might consider these services to provide a more holistic view of the challenges faced by SA groups.

### 4.2 Implications

These findings suggest that healthcare policies during crises should focus on cohesive and comprehensive primary health care surveillance systems. Such systems can help identify and address disparities more effectively, ensuring that vulnerable groups receive equitable healthcare access during pandemics to prevent the exacerbation of existing disparities. Effective pandemic responses such as preemptive planning and resource allocation could ensure that vulnerable groups are not disproportionately affected, benefiting both segregated and complementary populations. The results underscore the importance of monitoring and adjusting for health needs and status in future studies to better understand healthcare utilization patterns and inform more effective interventions.

## 5 Conclusion

This research investigated the impact of Hungarian COVID-19-related restrictions on healthcare utilization and outcomes among segregated and complementary populations. Despite significant reductions in healthcare use and reimbursement in 2020 compared with 2019, these changes did not disproportionately affect segregated populations, highlighting a highly stable Hungarian healthcare system. Instead, the observed increase in mortality appears to be linked to SES-based inequalities in the implementation of pandemic control measures, suggesting that these inequalities, rather than changes in healthcare access, contributed to the socioeconomic gradient in COVID-19 mortality. While these findings are not in line with the reported international experience, they underscore the importance of maintaining and equitable healthcare policies and targeted interventions to address existing disparities and ensure consistent access to healthcare services and, more importantly, preventive measures during crises.

## Data Availability

The raw data supporting the conclusions of this article will be made available by the authors, without undue reservation.
