# Supplementary material for: COVID-19’s effect on healthcare disparities: delivery, reimbursement, and premature mortality in residentially segregated populations
Source: Front Public Health. 2025 May 13;13:1481814. doi: 10.3389/fpubh.2025.1481814 (PMC12106325; doi:10.3389/fpubh.2025.1481814)
Supplement: Supplementary file 1 [file Table_1.docx]

Supplementary Material

## Supplementary Table

| Indicator name | Year | Observed cases in SA | Expected cases in SA | Standardized risk ratios for SA | Observed cases in CA | Expected cases in CA | Standardized risk ratios for CA | Relative Risk ratio |
| --- | --- | --- | --- | --- | --- | --- | --- | --- |
| Number of GP visits | 2019 | 2225524 | 1798041.71 | 1.238 | 47733596 | 48250227.7 | 0.989 | 1.251 |
|  | 2020 | 1993344 | 1609767.91 | 1.238 | 45760688 | 46233094.37 | 0.990 | 1.251 |
| Outpatient service use | 2019 | 186065 | 193696.31 | 0.961 | 4994363 | 4984278.22 | 1.002 | 0.959 |
|  | 2020 | 150414 | 158191.05 | 0.951 | 4372562 | 4359759.08 | 1.003 | 0.948 |
| Number of MRI/CT use | 2019 | 19264 | 20568.16 | 0.937 | 581321 | 578795.55 | 1.004 | 0.933 |
|  | 2020 | 15078 | 16042.48 | 0.940 | 478488 | 476047.03 | 1.005 | 0.935 |
| Number of Hospitalizations | 2019 | 46882 | 39098.76 | 1.199 | 1047340 | 1054452.36 | 1.006 | 0.890 |
|  | 2020 | 35527 | 28633.02 | 1.241 | 801291 | 807568.96 | 0.993 | 1.207 |
| Outpatient service reimbursement | 2019 | 4390.9 | 4902.99 | 0.896 | 135320.6 | 134450.58 | 1.006 | 0.890 |
|  | 2020 | 3418.39 | 3863.84 | 0.885 | 110613.69 | 109716.4 | 1.008 | 0.878 |
| MRI/CT reimbursement | 2019 | 716.49 | 870.2 | 0.823 | 24808.29 | 24605.82 | 1.008 | 0.817 |
|  | 2020 | 613.78 | 746.12 | 0.823 | 22293.1 | 22092.17 | 1.009 | 0.815 |
| Hospitalization reimbursement | 2019 | 14474.02 | 13972.39 | 1.036 | 404564.2 | 404847.79 | 0.999 | 1.037 |
|  | 2020 | 11362.72 | 10695.75 | 1.062 | 321744.68 | 321974.83 | 0.999 | 1.063 |
| Medication reimbursement | 2019 | 12912.22 | 15066.21 | 0.857 | 377076.08 | 374988.26 | 1.006 | 0.852 |
|  | 2020 | 12587.41 | 14448.45 | 0.871 | 380766.35 | 379755.5 | 1.003 | 0.869 |
| Premature mortality | 2019 | 1225 | 1054.45 | 1.162 | 19780 | 19959.57 | 0.991 | 1.172 |
|  | 2020 | 1208 | 1110.55 | 1.088 | 20313 | 20440.9 | 0.994 | 1.095 |

**Supplementary Table S1**. Observed and Expected Healthcare Utilization and Reimbursement Measures for SAs and CAs, with Standardized Risk Ratios in Hungary (2019-2020)
